# Autism Spectrum Disorder May Be Highly Prevalent in People with Functional Neurological Disorders

**DOI:** 10.3390/jcm12010299

**Published:** 2022-12-30

**Authors:** Belén González-Herrero, Francesca Morgante, Javier Pagonabarraga, Biba Stanton, Mark J. Edwards

**Affiliations:** 1 Neurosciences Research Centre, Molecular and Clinical Sciences Institute, St George’s University of London, London SW17 0RE, UK; 2 Departamento de Medicina, Universidad Autónoma de Barcelona (UAB), 08193 Bellaterra, Spain; 3 Neurology Department, King’s College Hospital, London SE5 9RS, UK; 4 Dipartimento di Medicina Clinica e Sperimentale, University of Messina, 98122 Messina, Italy; 5 Instituto de Investigación Biomédica de Sant Pau, 08041 Barcelona, Spain; 6 Centro de Investigación en Red-Enfermedades Neurodegenerativas (CIBERNED), 28031 Madrid, Spain; 7 South London and Maudsley NHS Foundation Trust, London SE5 8AZ, UK; 8 Department of Clinical and Basic Neuroscience, Institute of Psychiatry, Psychology and Neuroscience, King’s College London, London SE5 8AF, UK

**Keywords:** functional neurological disorders, autism spectrum disorder, prevalence, autistic traits, co-occurrence

## Abstract

Recent observations suggest that autism spectrum disorder (ASD) co-occurs in people with a functional neurological disorder (FND), but little systematic data are available on the relationship between FND and autism. The study aimed to assess the self-reported autistic traits via a standardized questionnaire and the prevalence of previously diagnosed ASD among people with FND and their 1st-degree relatives. We performed a survey of members of the patient organization FNDHope, using a self-completed questionnaire for screening for autistic traits and ASD: the adult autism subthreshold spectrum (AdAS spectrum). There were 344 respondents diagnosed with FND with a mean age of 39.8 ± 11.6 years (female sex 90%). Eight per cent of respondents volunteered a previous diagnosis of ASD, and 24% reported a 1st-degree relative with a formal diagnosis of ASD, mostly their children. We found that 69% of respondents had scores in the AdAS spectrum indicating a clinically significant ASD and 21% indicating autistic traits. Further studies are needed to provide more evidence regarding the prevalence of ASD in people with FND and how this may influence the aetiology, treatment selection and prognosis.

## 1. Introduction

Functional neurological disorder (FND) is a common neurological condition where the primary problem appears to be a genuine lack of voluntary access to movement and/or sensation, despite normal basic neurological function. People with FND most commonly present with abnormalities of movement control (e.g., weakness, speech difficulties, tremor and dystonic posturing), sensory and cognitive disturbances or attacks that resemble epilepsy (dissociative seizures/non-epileptic attacks). This frequently causes disability and a poor quality of life [1], and most people remain with symptoms in the long term [2]. Females are significantly more affected than males (2–3:1) in children and adults [3,4]. The symptom onset is usually in middle adulthood (mean age is 40 years [5]) but can affect children as young as six and older adults [6,7].

The aetiology of FND is not fully understood. Data suggest various risks, precipitant, and perpetuating factors. Previous stressful life events, including childhood or adult-life abuse, certain personality traits, anxiety and depressive disorders are significantly more common in patients with FND, compared to healthy and patient controls [8,9]. However, such predisposing factors are also absent in many people with FND.

Frequently, people with FND also present with functional somatic syndromes (FSSs), such as irritable bowel syndrome (IBS), fibromyalgia/widespread chronic pain (FM/CWP) or chronic fatigue syndrome (CFS) [10]. Despite these being listed as separate conditions in DSM-5, growing voices advocate for overlapping the clinical and aetiological characteristics between FSSs themselves but also with FND [11,12,13,14].

Some emerging but inconclusive data show neurodevelopmental differences in people with FND and FSSs, especially autism spectrum disorder (ASD). In a cohort of 288 patients with FND, 9% were found to have co-existing neurodevelopmental conditions, with a higher frequency seen in those with dissociative seizures [15]. Another study with 59 children with dissociative seizures found that 17% had ASD [16]. A longitudinal study of 77 women diagnosed with ASD/ADHD in childhood, found common co-existing chronic pain syndrome (77%) [17]. FMR1 gene premutation carriers, a well-known risk factor for neurodevelopmental differences, commonly report chronic pain symptoms [18].

Understanding if neurodevelopmental differences (especially ASD) are part of the pathophysiology of FND could lead to important insights into the aetiology and treatment. We hypothesized that autistic traits would be common among people with FND and assessed this using a validated self-completed questionnaire. We also studied the prevalence among people with FND and in their 1st-degree relatives of previous neurodevelopmental conditions with a special focus on ASD.

## 2. Methods

In this cross-sectional observational study, we administered an online survey to subjects who reported a diagnosis of FND received from a medical professional.

Recruitment was achieved by disseminating the questionnaire through the patient support groups FND Hope UK and the FND Hope International website and social media to individuals who had consented to be contacted with information of this sort. In total, these accounts have more than 7200 followers. The research protocol included some questions for gathering the sociodemographic data, and the invitation included information that neurodevelopmental differences, including ASD and their possible relationship to FND, were the topic of the study. Autistic traits were assessed by an online English version of the adult autism subthreshold spectrum (AdAS Spectrum) self-reported instrument developed and validated by Dell’Osso et al. [19].

The AdAS spectrum has 160 dichotomous items (yes/no). It is organized into seven domains: childhood/adolescence (21 items), verbal communication (18 items), non-verbal communication (28 items), empathy (12 items), inflexibility and adherence to routine (43 items), restrictive interests and rumination (21 items); hyper-hypo reactivity to sensory input (17 items). Its design best captures the new DSM-5 criteria referring, not only to the core manifestations of ASD, but also to the attenuated and atypical symptoms, the personality traits, and the behavioral manifestations that may be present in partial forms [19]. Further, more attention was given during its design to the female phenotypes of ASD and the sensory reactivity area of symptoms [20]. The domain and total scores are obtained by counting the number of positive answers, with higher scores indicating higher autistic traits. A cut-off of 43 to 69 points identifies an individual with autistic traits and 70 points or more as ASD [21].

A version of the AdAS spectrum was created via the Microsoft form platform and shared open access over a period of 6 weeks from 15 November to 27 December 2021. The data were returned in an anonymized fashion without any personal identifiers. The respondents were also asked about basic demographics, a previous medical professional diagnosis of neurodevelopmental conditions, such as autism spectrum disorder (ASD), attention-deficit/hyperactivity disorder (ADHD), learning disabilities (LDs), intellectual disability (ID), and diagnosis of these conditions in 1st-degree relatives.

## 3. Statistical Analysis

As a preliminary step, the cases with high levels of missing data in the AdAS spectrum (>10% in the same domain or the whole questionnaire) were removed. To account for the non-responded items, the total AdAS spectrum score and domain scores were weighted for the number of valid answers. The weighted score was calculated as the sum score multiplied by 160 (number of items in the questionnaire) and divided by the number of completed items. Item responses were preliminarily tested for asymmetry and kurtosis. Age was calculated as the year of birth to the survey year (2021). Descriptive statistics (i.e., frequencies, proportions/percentages, measures of central tendency and dispersion) were used to summarize the data. Averages are presented as mean ± standard deviation (SD). Group comparisons were performed using xi2 for the categorical variables and the *t*-test, Kruskal–Wallis and Mann–Whitney tests for the continuous variables, as required. The results were considered statistically significant at a 2-tailed *p* < 0.05. A post-hoc Bonferroni correction was applied for multiple comparisons, when required. The statistical analysis was performed by IBM SPSS Statistics 26.

## 4. Results

### 4.1. Demographics

The FND Hope UK and FND Hope International social media have approximately 7200 followers. The total number of people who saw the post is unknown, however, 361 people submitted responses. The study was open for six weeks, with 80% of the responses gathered during the first week of publication. This suggests the study was less visible over the following weeks when several invitations to the studies from other groups were published on subsequent days.

Of the total participants, 359 consented for their answers to be analyzed. Fourteen participants were directly excluded as they provided incomplete demographic data or had a significant number of responses missing from the questionnaire. One participant was excluded due to not having a formal diagnosis of FND. A total of 344 participants with FND were analyzed.

The data are presented in Table 1 and Table 2. Two hundred thirty-two respondents (67.4%) were based in the UK. The sex of the respondents was mostly female (*n* = 310, 90.1%, *p* < 0.001) with female gender identity (*n* = 298, 86.6%, *p* < 0.001) and had a mean age of 39.8 ± 11.6 years. A previous diagnosis of ASD was present in 27 (7.8%), ADHD in 23 (6.7%), and LD/ID were present in 23 (6.7%). Of the 143 (41.5%) participants that declared having a 1st-degree relative with a neurodevelopmental disorder, ASD was significantly more common (24%), followed by ADHD (11.6%) and LD/ID (5.8%), *p* < 0.001. Of those declaring having a first-degree relative with ASD, this was their children in 69.9%, *p* < 0.001.

Among those who indicated that they had children with a neurodevelopmental condition (*n* = 91, 26.5%), we asked whether the other parent was also diagnosed. This question was answered positively by 13 (14.2%), mostly accounted for ADHD (*n* = 7, 7.6%) and less frequently for ASD (*n* = 3, 3.2%) or learning disabilities (*n* = 3, 3.2%).

### 4.2. The AdAS Spectrum Questionnaire

The AdAS spectrum questionnaire data are summarized in Table 3. The total score on the AdAS spectrum questionnaire categorized the sample into three groups: group A (33, 9.6%) with respondents below the cut-off for the subthreshold autistic traits; group B (73, 21.2%), self-reporting significant subthreshold autistic traits; and group C (238, 69.2%) scoring in the range suggesting a clinically significant ASD (*p* < 0.001). Scores in the AdAS spectrum indicating a clinically significant ASD was related to the previous diagnosis of neurodevelopmental disorders (*p* = 0.003). Accordingly, the questionnaire classified all participants who reported a previous diagnosis of ASD, among the ASD spectrum (*n* = 2 group B, *n* = 25 group C). However, higher scores in the AdAS spectrum were not related to the diagnosis of neurodevelopmental disorders in first-degree relatives. All 12 respondents with different gender identities from female/male were classified in group C (clinically significant ASD).

Group C showed a mean age significantly lower, when compared with both group B (*p* = 0.005) and group A (*p* = 0.006). There were no significant age differences between groups A and B (*p* = 0.4).

The responders in all groups had higher scores in the inflexibility and non-verbal communication domains, and the low empathy domain score was the lowest in all groups. As the number of questions in each domain is different, when we performed the weighted scores for the number of questions in each domain, the responders in groups B and C had higher weighted scores in the childhood/adolescence and reactivity to sensory input domains, followed by verbal communication and restrictive interests. The low empathy and inflexibility weighted scores were the lowest in all groups.

## 5. Discussion

Here, we report the findings of a self-completed questionnaire screening for autistic traits and ASD in a cohort of 344 people with FND. We found that 69% of respondents had scores indicating a clinically significant ASD, and 21% with scores indicating autistic traits. Eight per cent of respondents volunteered a previous diagnosis of ASD, and 24% reported a 1st-degree relative with a formal diagnosis of ASD. Community-based prevalence rates for autism in the general population show considerable variation across studies, though recent systematic reviews and large-scale epidemiological research estimate rates of between 0.7–1.1% [22,23]. Studies in the psychiatric population show a variable prevalence from 2.4–9.9% [24]. Taken together, these data appear to indicate that ASD may be frequent in people with FND and their first-degree relatives.

Neurodevelopmental conditions are characterized by differences in cognition, communication, behavior and motor skills resulting from a unique brain development [25]. Learning disabilities (LDs), intellectual disability (ID), autism spectrum disorder (ASD), and attention-deficit/hyperactivity disorder (ADHD) all fall under this umbrella, with studies showing a substantial clinical heterogeneity and symptoms overlap [26,27,28]. ASD is commonly used to describe individuals with “core” difficulties in social, behaviour and communication domains. There is a broad range of severity, and some, particularly women, may have few “classic” autistic symptoms [29].

Some experimental data have previously suggested that key pathophysiological factors in individuals with FND are also present in people with ASD. For example, people with FND have alterations in attention allocation [30], emotional processing and alexithymia [31] and a sense of agency [32]. People with ASD also experience difficulties recognizing emotions, attention differences and a reduced sense of agency [33]. Studies of interoception, the neural network that senses changes in the body’s internal state and is strongly linked to emotional processing, show similar differences in people with ASD and FND [34,35,36].

However, contrasting data have also been published. No differences were found in the autism-spectrum quotient (AQ) score between a chronic fatigue syndrome group and a healthy control group, and both scored lower on the AQ than the group with ASD [37]. Studies on the “theory of mind”—the ability to attribute mental states to others and thought to be atypical in ASD—have reported no alterations in the “reading the mind in the eyes task” (RMET) in people with FND [31]. Stonnington et al. assessed the different theory of mind tasks in people with FND and medical controls, finding no differences between groups in most tasks, including the RMET [38]. These data should be cautiously interpreted however, as deficits in the theory of mind have been questioned in ASD [39], and the RMET is influenced by sex (higher scores in women), age, IQ and verbal skills [40].

We used the AdAS spectrum to assess the lifetime autistic traits [20]. Although alternative dimensional tools have been previously developed for measuring autistic traits amongst adults, such as the autism spectrum quotient (AQ) and the Ritvo Autism and Asperger’s diagnostic scale (RAADS) [41,42], the AdAS spectrum is the only one developed after the publication of the DSM-5 to assess the presence of the entire range of autism spectrum manifestations included in the new criteria. The AdAS spectrum questionnaire was designed to account for the female-specific manifestations of ASD/autistic traits. Functional neurological disorders are much more common in females than in males, and therefore, the use of a questionnaire designed considering the sex-related nuances of the ASD profile seemed the most appropriate for the aim of our study. The AdAS spectrum has been validated for the quantitative assessment of the autistic traits, reporting an excellent reliability (Kuder–Richardson’s coefficient of 0.964 for the total score) and a good convergent validity with the AQ (Pearson’s r correlation = 0.77) and the RAADS (Pearson’s r correlation = 0.83) [19]. A Spanish version has been validated [43] and used in several studies in the last few years [44,45,46,47,48,49].

The threshold values were established in a study including three groups: ASD, subjects with autistic traits, and healthy controls. Trained psychiatrists made the diagnosis, according to DSM-5, and the subjects self-completed the AdAS spectrum and the AQ. The best threshold value for identifying ASD was a score of 70, which showed a good agreement with the diagnosis performed, according to the DSM-5 criteria (specificity of 0.910 and sensibility of 0.923) and 43 for the autistic traits (specificity of 0.833 and sensitivity of 0.849) [21]. In the same study, the AQ had lower levels of agreement with the clinical evaluations, particularly in determining the presence of autistic traits. In our study, all participants who volunteered a previous diagnosis of ASD scored above the autistic trait or the ASD cut-off and, in general, had significantly higher total scores than those who did not report a previous ASD diagnosis.

Approximately 25% of respondents reported having a 1st degree relative with ASD, mainly their children (*n* = 58, 16.9%). The population variation in ASD occurrence is strongly related to genetic influences, with an estimated heritability of 80% [50]. Given that many respondents who reported relatives with ASD did not have a formal diagnosis themselves (despite their high scores on the AdAS spectrum), it may be that they have a subclinical or milder ASD phenotype than their relatives. This hypothesis is further supported by the female protective effect theory, which suggests that females (who are the majority of those affected by FND and the majority in our survey population) require a greater genetic “hit” than males to express the same degree of autistic characteristics [51,52,53], but also may be undiagnosed despite the significant impact from their autism due to the tendency of the diagnostic and screening processes to focus on characteristics that are most often seen in males with autism.

Obsessionality is a common feature among people with ASD [54]. Therefore, it is also interesting that previous studies have identified obsessive-compulsive personality traits and disorder to be more common in people with FND [9]. It is an open question whether co-existing ASD could explain these previous findings in people with FND that were not identified but led to positive scores on questionnaires for obsessive-compulsive personality disorder.

Why individuals with autism may have an increased vulnerability to developing FND, is still an unanswered question. Characteristics of the brain structure, function and connectivity may play a role, but there may also be a higher exposure to known risk factors for FND [8,9]. Exposure to early-life traumatic events, such as bullying and ostracism, is common in ASD [55], and differences in emotional processing and potentially in the family environment, if a parent also has autistic traits or ASD, may exacerbate and perpetuate the impact of the trauma on the emotional and personality development. In addition, the characteristics of some people with ASD, such as mental rigidity and information processing/perception differences, may alter the impact of even objectively mild-moderately challenging life experiences, affecting both the manifestation and severity of post-traumatic symptoms [56]. Conversely, it could be that the effects of early life trauma result in changes in personality, emotional processing and social interactions that mimic those seen in ASD, at least with regard to how a person may answer a questionnaire, such as the AdAS spectrum.

There are key links between the pathophysiology of ASD and FND in attentional processing, sense of agency and interoceptive processing. While the way in which these interact in both ASD and FND is still incompletely understood, the overlap of these features is intriguing. A key piece of missing understanding is why, despite some core underlying pathophysiological similarities, only a proportion of people with ASD develop FND and why this occurs typically in the 3rd to 4th decade, despite the lifelong nature of ASD.

## 6. Limitations

We acknowledge a number of limitations to our study. Almost 70% of people with FND in our sample scored in the range suggesting autism spectrum. Although we have anecdotally recognized several individuals in our clinics with probable ASD, this extremely high prevalence was surprising to us and makes it important to consider potential sources of bias in our data. We performed an online survey of a community with over 7200 members and gained only 361 respondents. Specific data on ethnicity, socioeconomic status and educational attainment were not recorded. There are, therefore, several possible biases here. First, the people who are part of the FND Hope online community may not be fully representative of all people with FND. Second, there may well have been a response bias for those with a specific interest in neurodevelopmental disorders or ASD. Online surveys, such as this, tend to favour younger patients and those with computer skills and may fail to reach those with poorer educational levels or more social deprivation. Although we chose a validated instrument to measure the autistic traits and ASD, this is not a substitute for a formal in-person interview and multidisciplinary assessment, and we cannot exclude that participants misunderstood or otherwise failed to give an accurate answer to the questions.

## 7. Conclusions

Our results revealed a high prevalence of people with FND who scored above the threshold for autistic traits and ASD on a validated self-completed questionnaire and who have a previous diagnosis of a neurodevelopmental disorder or 1st-degree relatives with neurodevelopmental disorders.

Further research is needed to understand the intersection between autism and FND. This is particularly important when approaching the FND diagnosis and delivering treatment, as people with autistic traits and ASD may have specific needs that require to be identified and properly integrated into treatment.

## Figures and Tables

**Table 1:** Demographic data.

Total *n* = 344	*n* (%)	*p*
**Sex**		<0.001
Female	310 (90.1)	
Male	32 (9.3)	
Intersex	2 (0.6)	
**Gender identity**		<0.001
Female	298 (86.6)	
Male	33 (9.6)	
Transgender	3 (0.9)	
Nonbinary	7 (2.0)	
Gender fluid	2 (0.6)	
Prefer not to say	1 (0.3)	
**Age (Mean ± SD, year)**		0.014
Female	39.4 ± 11.6	
Male	44.2 ± 11.1	
Intersex	19.9 ± 7.9	

*n* = number of respondents, SD = standard deviation. *p* = significant difference between groups is considered if *p* < 0.016 or <0.008 (post hoc Bonferroni correction for the multiple comparisons).

**Table 2:** Neurodevelopmental disorders data in the respondents and their first-degree relatives.

	Total *n* (%)	ASD *n* (%)	ADHD *n* (%)	LDs/IDs *n* (%)	*p*
**Previous diagnosis of NDDs**	73 (21.2)	27 (7.8)	23 (6.7)	23 (6.7)	0.8
**NDDs 1st-degree relatives**	143 (41.6)	83 (24.1)	40 (11.6)	20 (5.8)	<0.001
Children		58 (16.9)	22 (6.4)	11 (3.2)	<0.001
Siblings		22 (6.4)	16 (4.7)	6 (1.7)	<0.001
Parents		3 (0.9)	2 (0.6)	3 (0.9)	0.8

*n* = number of respondents, SD = standard deviation, NDDs = neurodevelopmental disorders, ASD = autism spectrum disorder, ADHD = attention-deficit/hyperactivity disorder, LDs = learning disabilities, IDs= intellectual disabilities. *p* = significant difference between groups is considered if *p* < 0.016 or <0.008 (post hoc Bonferroni correction for multiple comparisons).

**Table 3:** Classification of the respondents according to the adult autism subthreshold spectrum (AdAS spectrum score) and comparison of the self-reported scores in the AdAS spectrum total score and subdomains.

Total *n* = 344	Group A Cut-off <43	Group B Cut-off ≥43–<70	Group C Cut-off ≥70	*p*
**Total participants, *n* (%)**	33 (9.6)	73 (21.2)	238 (69.2)	<0.001
**Age, Mean (SD), yrs**	43.9 ± 9.9	42.7 ± 11.2	38.2 ± 11.7	<0.05 *
**Diagnosis of NNDs, *n* (%)**				
No	28 (8.1)	66 (19.2)	177 (51.5)	<0.001
Yes	5 (1.5)	7 (2.0)	61 (17.7)	<0.001
**Total Score, Mean (SD)**	29.7 ± 10.7	58.9 ± 7.3	98.5 ± 17.8	<0.001
**Domains, Mean (SD) Scores**				
Childhood/Adolescence	5.1 ± 2.9	10 ± 3.1	14.1 ± 3.0	<0.001
Verbal Communication	3.8 ± 2.8	7.3 ± 2.5	11.5 ± 3.1	<0.001
Non-Verbal Communication	6 ± 3.2	10.7 ±3.2	16.1 ± 3.9	<0.001
Low Empathy	1.2 ± 1.4	3.3 ±2.1	6.9 ± 2.7	<0.001
Inflexibility and Adherence to Routine	6 ± 3.6	14.3 ± 4.2	25.4 ± 6.4	<0.001
Restricted Interests and Rumination	3.4 ± 2.2	7.3 ± 2.2	13.4 ± 3.5	<0.001
Hyper- and Hyporeactivity to Sensory Input	4.3 ± 2.1	6.95 ± 2.7	11.0 ± 2.9	<0.001

Data presented in the table correspond to the self-reported scores in The AdAS spectrum score. Participants are classified as group A (below the cut-off for subthreshold autistic traits), group B (significant subthreshold autistic traits) and group C (clinically significant ASD) according to the total score. NDDs = neurodevelopmental disorders, ASD = autism spectrum disorder, ADHD = attention-deficit/hyperactivity disorder, LDs = learning disabilities, IDs = intellectual disabilities. *n* = number of respondents. % = percentage of the total respondents. *P* = significant difference between groups is considered if *p* < 0.05 or *p* < 0.016 when appropriate (post hoc Bonferroni correction for multiple comparisons). * Group C showed a mean age significantly lower, when compared with both group B (*p* = 0.005) and group A (*p* = 0.006). There were no significant age differences between groups A and B (*p* = 0.4).

## Data Availability

Supporting data is not publicly available.
